# Clinical Manifestations in a Girl with NAA10-Related Syndrome and Genotype–Phenotype Correlation in Females

**DOI:** 10.3390/genes12060900

**Published:** 2021-06-10

**Authors:** Ilenia Maini, Stefano G. Caraffi, Francesca Peluso, Lara Valeri, Davide Nicoli, Steven Laurie, Chiara Baldo, Orsetta Zuffardi, Livia Garavelli

**Affiliations:** 1 Child Neuropsychiatry Unit, Azienda USL di Parma, 43121 Parma, Italy; imaini@ausl.pr.it; 2 Medical Genetics Unit, Azienda USL-IRCCS di Reggio Emilia, 42123 Reggio Emilia, Italy; StefanoGiuseppe.Caraffi@ausl.re.it (S.G.C.); francesca.peluso@ausl.re.it (F.P.); lara.valeri@ausl.re.it (L.V.); 3 Post Graduate School of Paediatrics, University of Modena and Reggio Emilia, 41124 Modena, Italy; 4 Molecular Biology Laboratory, Azienda USL-IRCCS di Reggio Emilia, 42123 Reggio Emilia, Italy; Davide.Nicoli@ausl.re.it; 5 Clinical Genomics, Centre Nacional d’Anàlisi Genòmica, Centre de Regulació Genòmica, 08016 Barcelona, Spain; steven.laurie@cnag.crg.eu; 6 Laboratory of Human Genetics, Galliera Hospital, 16128 Genoa, Italy; chiarabaldo@gaslini.org; 7 Unit of Medical Genetics, Department of Molecular Medicine, University of Pavia, 27100 Pavia, Italy; orsetta.zuffardi@unipv.it

**Keywords:** *NAA10*-related syndrome, X-linked disorder, syndromic and non-syndromic intellectual disability, genotype–phenotype correlation

## Abstract

Since 2011, eight males with an X-linked recessive disorder (Ogden syndrome, MIM #300855) associated with the same missense variant p.(Ser37Pro) in the *NAA10* gene have been described. After the advent of whole exome sequencing, many *NAA10* variants have been reported as causative of syndromic or non-syndromic intellectual disability in both males and females. The *NAA10* gene lies in the Xq28 region and encodes the catalytic subunit of the major N-terminal acetyltransferase complex NatA, which acetylates almost half the human proteome. Here, we present a young female carrying a de novo *NAA10* [NM_003491:c.247C > T, p.(Arg83Cys)] variant. The 18-year-old girl has severely delayed motor and language development, autistic traits, postnatal growth failure, facial dysmorphisms, interventricular septal defect, neuroimaging anomalies and epilepsy. Our attempt is to expand and compare genotype–phenotype correlation in females with *NAA10*-related syndrome. A detailed clinical description could have relevant consequences for the clinical management of known and newly identified individuals.

## 1. Introduction

*NAA10*-related syndrome is an X-linked condition associated with defects in the N-alpha-acetyltransferase 10 (*NAA10*) gene. Clinical features range from a severe phenotype in males, originally described as Ogden syndrome and associated with the missense variant p.Ser37Pro [1], to a milder *NAA10*-related intellectual disability (ID) caused by different variants in both males and females [2]. Boys affected by Ogden syndrome had severe developmental delay, hypotonia, craniofacial abnormalities, cardiac anomalies, and early death in the first months of life, mainly due to cardiac arrythmias [1]. *NAA10* encodes the catalytic subunit of the N-terminal acetyltransferase protein complex A (NatA), responsible for acetylating 40–50% of all human proteins [3]. N-terminal (Nt) acetylation is a common protein modification in eukaryotes that occurs during protein synthesis and involves the transfer of an acetyl group from acetyl-coenzyme A to the protein alpha-amino group [4]. Molecular investigations in Ogden syndrome patients revealed reduced NatA activity and reduced Nt-acetylation of NatA substrates [5]. Since then, several different non-lethal *NAA10* variants have been reported and the clinical spectrum related to N-terminal acetylation deficiency has broadened [6,7,8,9,10,11,12,13]. In the past few years, NatA relevance to cardiac function [14], blood pressure regulation [15], developing brain [16], neurodegenerative disease [17,18], cancer, and cell cycle progression [19,20] has been described. *NAA10* has a universal expression pattern in human fetal and adult tissue. For its ubiquitous nature and its role in modifying this extent of the human proteome, NatA dysregulation can have extremely variable and profound consequences. Different variants in *NAA10* lead to various degrees of impairment in the enzymatic activity of NatA, but also specifically affect properties of *NAA10* function contributing to the wide phenotypic spectrum [8,9,13,21].

Hemizygous males with *NAA10*-related syndrome can attain clinical attention at different ages. They may show pre-natal cardiac manifestations (arrhythmias or congenital heart defects) or they may present at birth with cardiac concerns, hypotonia, brain abnormalities and dysmorphic features. Otherwise they may reveal relatively non-specific features later in childhood, such as developmental delay, growth retardation, skeletal findings, redundancy or laxity of the skin, cutaneous capillary malformations, genitourinary anomalies, and recurrent infections [2].

The phenotype of a heterozygous female with *NAA10*-related syndrome can range from asymptomatic to having some of the same clinical features as affected males, such as a variable degree of ID and cardiac findings. Varying female phenotypes depend not only on specific *NAA10* variants but also on X-chromosome skewing [2]. Only a few carrier mothers of males affected with Ogden syndrome have been diagnosed with ID or other severe medical conditions. In asymptomatic mothers, X-chromosome inactivation (XCI) in the blood was almost completely skewed toward the wild-type allele [5]. Casey et al. described the mother of two males with a p.Tyr43Ser variant showing learning disability, prolonged QTc interval and premature coronary artery disease. XCI studies detected a normal inactivation pattern in the blood [7].

Almost all clinical cases reported with *NAA10*-related syndrome carried *NAA10* missense variants [6,7,8,9,10,11,22]. Polyadenylation signal variants, resulting in a decreased quantity of *NAA10* RNA, seemed to be related to a distinct clinical phenotype in males, *NAA10*-associated syndromic microphthalmia [12,21]. Truncating variants in *NAA10* have been reported in just two cases, so it is possible that a complete loss of function might be embryonically lethal [2,8]. In both cases, males with severe developmental delay and microphthalmia in one individual, display hemizygous frameshift variants that leave the catalytic domain of *NAA10* intact, hypothetically leading to a residual level of truncated protein lacking important regulatory regions [13,23].

To date, 35 girls with *NAA10*-related syndrome due to 12 different *NAA10* missense variants have been reported [8,9,10,13,24,25,26]. We present an overview of these 12 *NAA10* variants with the corresponding phenotype and add our clinical case of a young female with syndromic ID carrying the most common de novo variant p.Arg83Cys (Table S1).

## 2. Materials and Methods

DNA samples of the trio, provided by the Biobank of the Laboratory of Human Genetics of IRCCS Gaslini (Genova, Italy), was analyzed by Whole Exome Sequencing (WES) as part of the project, “2016 BBMRI-LPC Whole Exome Sequencing call” (FP7/2007–2013) at the Centro Nacional de Análisis Genómico (CNAG-CRG) of Barcelona (Spain), as already described [27]. Presence of the NAA10 variant in the proband and its absence in her parents were confirmed by Sanger sequencing, with primers Forward: 5′-GGATCGGCTGCTCTGATCCA-3′ and Reverse: 5′-CCCCAGGACCCCCGTCAAGG-3′ (489nt amplicon).

## 3. Results

The proband of this case report is a girl, the second child of healthy, non-consanguineous parents. An examination of both parents and the older sister revealed a normal phenotype, and the mother was not exposed to any known teratogen during pregnancy. The patient was born at term by vaginal delivery at the 40th week, with a birth weight of 3575 g (50th percentile), and regular length and head circumference. Apgar scores were 8 at the 1st min and 10 at the 5th min. At birth she presented muscular ventricular septal defect (VSD), congenital hip dysplasia, generalized muscular hypotonia and dystonic movements.

**Figure 1:**
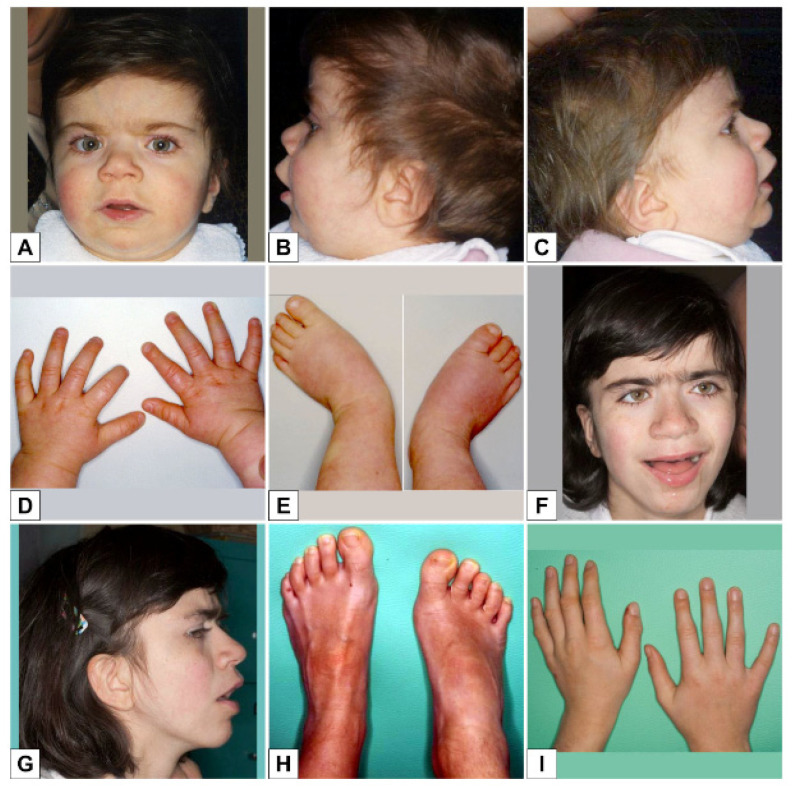
(**A**–**C**) Clinical features at the age of 1 year: mild coarse face, high-arched, thick eyebrows, long eyelashes, synophrys, broad nasal tip, anteverted nares, smooth long philtrum, thin upper lip, mild low-set ears, and hirsutism; (**D**,**E**) hands and feet; (**F**,**G**) clinical features at the age of 16 years: mild coarse face, high-arched thick eyebrows, long eyelashes, synophrys, broad nasal tip, anteverted nares, smooth long philtrum, thin upper lip, low-set ears and hirsutism; (**H**,**I**) hands and feet.

She came to the attention of our Medical Genetics Unit at 9 months of age; her head circumference was 43 cm (3rd percentile), her height was 69 cm (25th percentile) and her weight was 7380 g (3rd–10th percentile). Her pubertal stage was A0 B1 P1. Some facial dysmorphisms were noted: mild coarse face, bitemporal narrowing, arched thick eyebrows, long eyelashes, synophrys, broad nasal tip, anteverted nares, smooth long philtrum, thin upper lip, mild low-set ears, and hirsutism (Figure 1A–C). She had no significant hand or foot abnormalities (Figure 1D,E).

**Figure 2:**
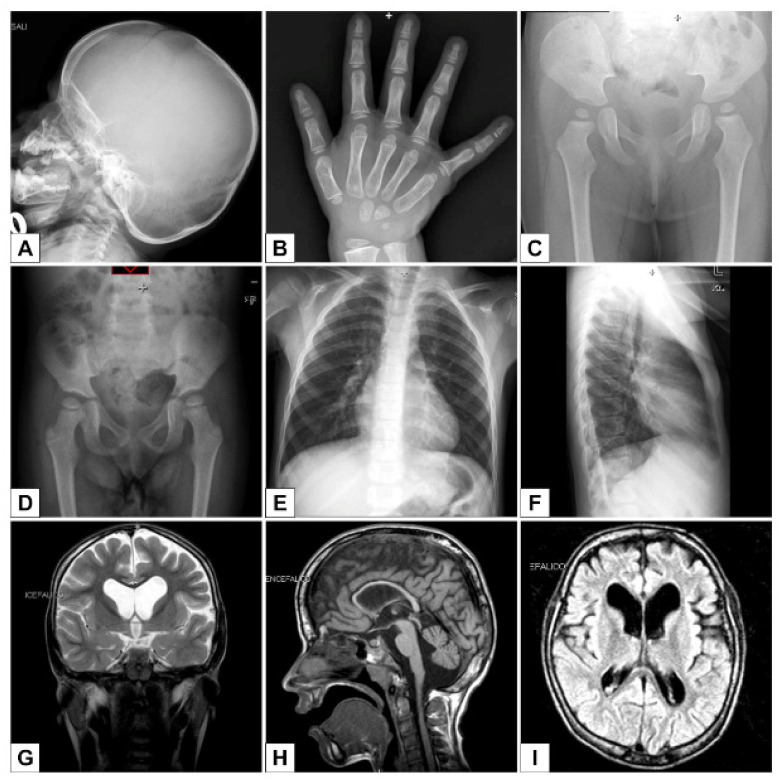
(**A**) Skull X-ray: large fontanel and Wormian bones; (**B**) Hand X-ray: delayed bone age. According to Greulich and Pyle Atlas at a chronological age of 4 years and 9 months, her bone age was consistent with an age of 2 years and 5 months; (**C**,**D**) Hip X-rays at 1 and 6 years: hip dysplasia; (**E**,**F**) Spine X-rays: scoliosis and mild thoracic kyphosis; (**G**–**I**) Brain MRI; (**G**) Coronal DWI image: large frontal horns of the lateral ventricles; (**H**) Sagittal T1 image: cerebellar atrophy and thin corpus callosum; (**I**) Axial FLAIR image: frontal lobes atrophy and large frontal horns of the lateral ventricles.

Her psychomotor development was significantly delayed: she got into sitting position at 18 months and she reached the standing position with help at 30 months; her speech remained limited to prelinguistic vocalization. Ophthalmologic investigation revealed myopia and astigmatism; fundus oculi showed slightly pale and broad appearance of the optic disc with central colobomatous defect. Audiometry test detected conductive hearing loss; auditory brainstem response was normal. A cardiac ultrasound confirmed apical muscular VSD and showed a mild to moderate hypertrophic cardiomyopathy. An abdominal ultrasound detected oversized spleen and liver, accessory spleen located at the level of the lower pole and left mild renal pyelectasis. Cranial X-ray demonstrated large fontanels and Wormian bones (Figure 2A). A hand X-ray showed delayed skeletal maturation: at a chronological age of 4 years and 9 months, her bones were consistent with an age of 2 years and 5 months, according to the Greulich and Pyle atlas (Figure 2B). Hip X-rays carried out at 1 and 6 years of age showed hip dysplasia (Figure 2C,D). X-ray of the spine at the age of 8 years demonstrated c-shaped scoliosis and mild thoracic kyphosis (Figure 2E,F).

Thyroid function tests, copper test and metabolic investigations bore normal results. Regarding neurological investigations, during the first years of life, EEG revealed abnormal brain network activity. She has been subject to epileptic seizures since the age of 6 years, and a diagnosis of Lennox-Gastaut Syndrome was made. Electroneurography detected a sensory neuropathy. A brain MRI showed frontal lobes and cerebellar atrophy, thin corpus callosum and dilation of the frontal horns of the lateral ventricles (Figure 2G–I).

At the last examination at 16 years of age, the patient showed severe growth delay: her head circumference was 52 cm (<3rd percentile; −2SD), height 138 cm (<3rd percentile; −3SD) and weight 34 kg (<3rd percentile). Her pubertal stage was A++ P4 B4. She had mid-face hypoplasia, thick eyebrows, synophrys, broad nasal tip, deep philtrum, thick lips, low-set ears (Figure 1F,G), deep palmar creases, flat feet, marbled skin, unsteady wide gait, axial hypotonia, dystonic movements (Figure 1H,I).

Currently, at 18 years of age, she has severe ID, can walk unaided only for short distances, and has absent speech. She shows frequent motor stereotypes such as rocking. She basically has a calm temperament but had a history of auto- and hetero-aggressive behaviour.

Karyotype was normal: 46,XX. Array-CGH showed a 227 kb microdeletion of paternal origin in the short arm of chromosome 3 [GRCh37 chr3p14.2(58,799,417-59,026,972)x1pat]. It was classified as a variant of uncertain significance, but it is probably unrelated to the patient’s phenotype since it does not contain any OMIM morbid genes, and the father was healthy. FISH telomere testing, FISH analysis for Angelman Syndrome and FISH analysis for Smith-Magenis Syndrome were normal. Molecular analysis of the *CDKL5, ARX, MERRF, FOXG1, MECP2, UBE3A* genes were normal. Molecular analysis of the *NIPBL* gene detected a benign variant of paternal origin (NM_133433.4:c.6764-35C>G). Whole exome sequencing (WES) revealed a heterozygous X-linked missense variant in the *NAA10* gene [NM_003491:c.247C>T, p.Arg83Cys]. This variant was not detected in either parent, thus arising de novo in the proband, and it has been previously reported as pathogenic in variant databases (ClinVar, HGMD).

## 4. Discussion

In recent years, after the advent of next-generation sequencing, several *NAA10* variants have been identified and the phenotypic spectrum associated with NatA deficiency affecting both males and females has greatly expanded [13].

In this report, we describe a girl with severely delayed motor and language development, autistic traits, facial dysmorphism, hypotonia, epilepsy, neuroimaging anomalies, VSD, postnatal growth failure, conductive hearing loss and skeletal abnormalities. WES identified the heterozygous de novo missense variant p.Ag83Cys in the *NAA10* gene.

To date, 35 girls with *NAA10*-related syndrome carrying 12 different *NAA10* missense variants have been described [8,9,10,13,24,25,26]. To our knowledge all these missense variants were identified as de novo alterations, with the exception of one maternal germline mosaicism.

From the comparison of clinical characteristics (Supplementary Table S1) we observed the presence of a neurodevelopmental disorder as the common feature in all cases. Variable degrees of neurodevelopmental disabilities included motor and language developmental delay, ID, learning impairment, autism spectrum disorder (ASD), attention deficit hyperactivity disorder and other behavioural issues. Furthermore, mild facial dysmorphism with no specific pattern, impaired motor function, and in particular, hypotonia, brain imaging anomalies, congenital cardiac anomalies, feeding difficulties and abnormality of the eyes were frequently described.

Functional studies about almost every variant have been performed and different effects on the overall NatA acetyltransferase activity and complex stability have been detected [8,9,10,13,24,25,26]. Table S1 highlights that p.Val111Gly and p.Arg116Trp variants [9,25], associated, respectively, with an unchanged NatA catalytic function and with a very mild reduction of NatA catalytic activity, result in milder clinical phenotypes, characterized by isolated ID or minor neurological and cardiac anomalies.

When compared to the nearly abolished catalytic activity associated with the p.Phe128Leu, p.Phe128Ile and p.Val107Phe variants, the partial catalytic impairment (about 60%) of the NAA10 subunit caused by the p.Arg83Cys variant was not reflected in different degrees of clinical phenotypes, suggesting the possibility of a critical threshold of enzyme activity necessary for normal function [9]. In subsequent functional studies on a recombinant NatA complex comprising both the NAA10 and the NAA15 subunits, Cheng et al. revealed that the *NAA10* p.Arg83Cys variant resulted in enhanced Nt-acetylation activity, suggesting that the phenotype of individuals with this variant might manifest through a different mechanism of action than other missense variants [13]. Nevertheless, the number of clinical cases reported for each *NAA10* variant identified is still too limited and a genotype–phenotype comparison does not yet appear to be significant, except for the p.Arg83Cys variant.

Indeed, the *NAA10* variant detected in our patient (p.Arg83Cys) is the most commonly reported and has been previously described in 19 female patients [9,10,13] (Supplementary Table S2). The common phenotype depicted for this subset consisted of severe developmental delay, resulting in moderate to severe ID with significant language impairment or absent speech and severe walking impairment, associated with ASD or autistic traits. Feeding difficulties, either temporarily in infancy or continuously with the need for tube feeding, and postnatal growth failure with final short stature were described in about two thirds of the patients. Variable degrees of visual impairment, including refractive disorder, strabismus, papillae abnormalities, and cortical visual impairment, were frequently reported. Facial dysmorphisms were present in more than half of the girls reported, especially coarse face, prominent forehead, bitemporal narrowing, arched eyebrows, and up-turned nose; nevertheless a clear dysmorphic pattern has not been identified. Microcephaly, hypotonia and brain imaging anomalies, such as white matter hypoplasia, thin corpus callosum and enlarged ventricles, were also common. Half of the girls described showed different degrees of congenital structural or conduction cardiac anomalies. Skeletal abnormalities, such as pectus excavatum and vertebral anomalies, were reported in some patients. Large fontanels have been previously described in three other girls, while delayed skeletal maturation has been reported only in our case, but apparently it was not tested in the others. Other clinical features such as hearing impairment, cutis marmorata, sleeping problems and seizures were less frequently described.

In addition to our patient, seizures were reported in 4 other girls with the same variant and in a total of 8 previously described girls carrying different *NAA10* variants. Clinical characteristics of seizures described were variable: focal onset dyscognitive seizures associated with apnea and secondary generalized seizures starting from 11 years of age [10], absence seizures, partial epilepsy, tonic–clonic seizures, myoclonus epilepsy with generalized sharp waves developing at the age of 12 years [13], myoclonic generalized seizures and bifrontal slow waves and spike waves [9].

In summary, our patient confirmed the main clinical features previously described in females with *NAA10*-related syndrome carrying the same common variant (p.Arg83Cys) and showed compatible but less frequent characteristics, such as epilepsy and conductive hearing loss. She also showed sensorial neuropathy that expands the *NAA10* clinical phenotype.

Our comparison (Supplementary Table S1) highlights that genotype–phenotype correlation for different *NAA10* variants is still far from being defined, because of the small number of patients described for each variant.

It is well established that the wide spectrum of clinical features in heterozygous females with *NAA10*-related syndrome, ranging from isolated mild ID to variable degrees of developmental disorders associated to different clinical anomalies, depends on specific variants, and presumed favorable versus non-favorable XCI. Nevertheless, XCI was not frequently investigated, making it difficult to speculate about its impact on the phenotypic expression of the disease.

## 5. Conclusions

The *NAA10*-related syndrome is characterized by wide phenotypic variability in both sexes, ranging from early lethality due to structural cardiac abnormalities and/or arrhythmias in males with the recurrent Ser37Pro variant, to a series of disorders reported in both males and females with other *NAA10* variants, such as developmental delay, muscle hypotonia, skeletal abnormalities, recurrent infections, feeding difficulties, postnatal growth retardation, dysmorphism, ocular anomalies and cardiac abnormalities, including long QT syndrome.

The manifestation of disease symptoms in females appears to be, at least in part, linked to the type of variant. Only the Ser37Pro variant, detected in mothers of males individuals affected by Ogden syndrome, appears to be completely asymptomatic. However, the assumption that it may be completely benign in females is highly suspicious, given the fact that it is not reported in gnomAD, despite its recurrence. The other *NAA10* variants, falling either in the same domain of Ser37Pro or throughout the gene, are associated with a huge series of phenotypic alterations, both in the mothers of males with more severe disease and in affected females in which the variant is de novo.

The presence of phenotypic alterations in carriers of X-linked recessive variants has traditionally been associated with skewed rather than random XCI, resulting in clusters of cells in which the active X chromosome is the one with the variant and thus partially mimics the condition of hemizygous males. To date, a direct correlation between the presence of clinical symptoms or their severity and the degree of skewed XCI is difficult if not impossible to assess, also due to the evident difficulty in examining other tissues besides blood. However, it seems logical to hypothesize that the final result of the mosaicism created by XCI follows the pattern of all mosaics i.e., it depends on a set of genomic variants affecting the whole genome [28,29]. The resulting polygenic score will define the risk that the active X chromosome is the one with the variant associated with the disease, presumably in a tissue-specific manner. Given the current inability to predict genotype–phenotype correlations based on XCI, functional analysis of *NAA10* variants and detailed clinical description of patients remain fundamental to understand how factors and mechanisms contribute to the broad clinical spectrum of disorders related to *NAA10*.

## Data Availability

The data that support the findings of this study are available from the corresponding author upon reasonable request.

## Supplementary Materials

The following are available online at https://www.mdpi.com/article/10.3390/genes12060900/s1, Table S1: Summary of molecular findings and clinical features in females with *NAA10* variants, Table S2 Summary of clinical features in females with the most common *NAA10* variant (*p*.Arg83Cys).
