# Supplementary material for: Clinical Manifestations in a Girl with NAA10-Related Syndrome and Genotype–Phenotype Correlation in Females
Source: Genes (Basel). 2021 Jun 10;12(6):900. doi: 10.3390/genes12060900 (PMC8230408; doi:10.3390/genes12060900)
Supplement: Supplementary file 1 [file genes-12-00900-s001.zip › genes-1252873-supplementary/genes-1252873-sippl/Supplementary Table S1 NAA10 01.6FP JMK SC.pdf]

Table S1. Summary of molecular findings and clinical features in females with *NAA10* variants.

**Reference**	**Cheng et al. 2019**	**Bader et al. 2020**	**Saunier et al. 2016, Sidhu et al. 2017, Cheng et al. 2019 Our case**	**Cheng et al. 2019**	**Cheng et al. 2019**	**Popp et al. 2015**	**McTiernan et al. 2018**	**Saunier et al. 2016**	**Cheng et al. 2019**	**Thevenon et al. 2016**	**Saunier et al. 2016, Cheng et al. 2019**	**Cheng et al. 2019**	**Total**
NAA10 mutation	**Leu11Arg**	**His16Pro**	**Arg83Cys**	**Ala87Ser**	**Ala104Asp**	**Val107Phe**	**Val111Gly**	**Arg116Trp**	**Leu121Val**	**Phe128Ile**	**Phe128Leu**	**Met147Thr**	**12**
CADD score	32	28.6	28.9	25.4	27.6	26.1	28.3	24.3	25.8	27.9	23.4	24.0	
Functional studies	reduction in NatA catalytic activity	impaired NatA complex formation	clear reduction (60%) in catalytic activity; enhance catalytic activity (Cheng et al. 2019)	NA	reduction in NatA catalytic activity	nearly abolished enzymatic activity	decreased stability, NatA catalytic activity unchanged	very mild reduction in catalytic activity	NA	decreased stability, near loss activity (>90%)	decreased stability, near loss activity (>90%)	very mild reduction in catalytic activity	
Patients	1	1	20	3	1	1	1	1	2	1	3	1	**36**
Inheritance	NA	*de novo*	*de novo* (19/20), MGM (1/20)	*de novo* (3/3)	*de novo*	*de novo*	*de novo*	*de novo*	*de novo* (1/2), NA (1/2)	*de novo*	*de novo*	*de novo*	*de novo* **(33/36)**
X-inactivation	NA	skewed	random (1/20), NA (17/20), 92% (1/20), 100% (1/20)	NA (3/3)	NA	random	random	random	NA(2/2)	random	random (1/3), NA (2/3)	NA	
Feeding difficulties			14/20	2/3	+				1/2		1/3	+	**20/36**
Short stature		+	10/20	1/3		+			1/2	+	2/3		17/36
Microcephaly		+	11/20			+					1/3	+	15/36
Facial Dysmorphism		+	14/20	2/3		+			1/2	+	2/3		**22/36**
NDD/ ID	+	+	20/20	3/3	+	+	+	+	2/2	+	3/3	+	**36/36**
Seizures			5/20	2/3							2/3		9/36
Muscular hypotonia		+	10/20	2/3		+		+	1/2	+	1/3		18/36
Neuropathy			1/20										1/36
Movement disorder			4/20	1/3	+						1/3		7/36
Brain imaging anomalies	+	+	9/20	2/3		+ (mild)		+ (thin CC, dilated LV)		+	3/3	+	**20/36**
NDD		autism	8/20 autism	1/3 (autism, ADHD)	autism, ADHD	autism		ADHD, DCD	1/2 autism				15/36

Behavioural abnormalities			7/20			+			1/2				9/36
Cardiac anoamlies			10/20			+		+		+	1/3		14/36
Eye abnormalities	+	+	14/20	1/3	+	+			1/2		1/3	+	**22/36**
Genitourinary system abnormalities			2/20										2/36
Skeletal system abnormalities		+	10/20	1/3	+	+ (large fontanels, delayed bone age)			1/2		1/3		16/36
Ear abnormalities			3/20	1/3	+								5/36
GI tract abnormalities			5/20										
Skin abnormalities			4/20										4/36
Other			sleep disorder		sleep disorder		bruxism						
