# Supplementary material for: Clinical Manifestations in a Girl with NAA10-Related Syndrome and Genotype–Phenotype Correlation in Females
Source: Genes (Basel). 2021 Jun 10;12(6):900. doi: 10.3390/genes12060900 (PMC8230408; doi:10.3390/genes12060900)
Supplement: Supplementary file 1 [file genes-12-00900-s001.zip › genes-1252873-supplementary/genes-1252873-sippl/Supplementary Table S2 NAA10 Arg83Cys 01.6.FP JMK con revisioni.pdf]

Table S2. Summary of clinical features in females with the most common *NAA10* variant (*p*.Arg83Cys).

	ID	Inheritance,X-inact.	Age	Facial Dysmorphism	micro-cephaly	Short stature	Neurodevelopment	hypo/ hypertonia	Brain imaging anomalies	Feeding difficulties	Cardiac anomalies	Eye abnormalities	Skeletal system abnormalities	Others
Saunier et al. 2016	1	De novo, random	4y 2mo	NA	-3.5 SD	yes	Severe DD, absent speech	hypo	no	yes	no	no	no	no
	2	de novo, 92%	4y 3mo	NA	-3.59 SD	yes	DD (not walking at 3y 6mo, severe language delay), aggressivity	hypo	hyppocampic dysgenesis	no	no	no	no	no
	3	de novo, NA	2y 2mo	prominent forehead, up-lifted ear lobes, narrow palate	-3.86 SD	yes	severe DD (not walking yet and absent speech)	hypo	periventricular white matter loss	yes	mild PAS, PFO vs. ASD	myopia, astigmatism	large fontanels, super-numerary vertebra, dysmorphic L1	no
	4	de novo, NA	3y 10mo	NA	-4.85 SD	yes	severe DD (not walking yet and absent speech)	hypo/ hyper	IVH occipital horn, PVL, HIE	yes	no	alternating esotropia, cortical visual impairment	large fontanels, small hands and feet, thoracic anomalies	VP shunt
	5	de novo, NA	10y6mo	bitemporal narrowing, arched eyebrows, synophrys, upturned nose, uplifted earlobes, hirsutism	-2.8 SD	yes	DD (walking at 3y), moderate ID, very active, problem in new sitting	hypo	no	mild	long QT	astigmatism, hyperopia	large fontanels, mild pectus excavatum	cutis marmorata
	6	de novo, 100%	4y	arched eyebrows, upturned nose	no	no	severe DD (walking at 2y, absent speech), hyperactivity, poor eye contact, aggressivity	no	NA	yes	incomplete right bundle branch block	hyperopia	clinodactily V, pectus excavatum	sleeping problem
	7	MGM, NA	6y 6mo	prominent forehead, bitemporal narrowing, arched eyebrows, upturned nose	-4.85 SD	yes	Severe ID (not walking yet and absent speech), attention deficit, restlessness	periph hyper	NA	NA	no	myopia, megalopapillae	small hands and feet, tapering fingers, pectus excavatum	cutis marmorata
Sidhu et al. 2017	8	de novo, NA	13y	frontal bossing, bitemporal narrowing, low set ears, coarse	NA	NA	DD (walking with assistance at 25mo), severe ID, autism spectrum disorder	hypo	white matter volume loss, thin CC, ventriculomegaly	no	no	no	broad big toes	seizures, hyper-somnolence

				face, high arched palate										
	9	de novo, NA	10y	no	no	yes	Global DD, severe ID, autistic traits, apraxia, poor fine motor skills	hypo/ hyper	no	functional bulbar palsy, esophagus and gut dysmotility syndrome, complete dysphagia	no	cortical visual impairment, bilateral astigmatism, divergent squint	kyphosis	severe sleep disorder, bilateral talipes, hypertrichosis
	10	de novo, NA	13y	coarse face, synophris, large nose	no	no	severe DD (very limited speech, poor fine motor skills)	no	white matter loss, thin CC, prominent CSF spaces	PEG feeding	long QT, bicuspide valve	astigmatism	pectus excavatum	absence seizures, premature precocious puberty
	11	de novo, NA	11y 6mo	micrognathia	no	no	global DD (poor fine motor skills, absent speech), ID, autism spectrum disorder	no	small cyst	PEG feeding, GI dismotility, cyclical vomiting, food intolerance	NA	cortical visual impairment	no	moderate hearing impairment, precocious puberty
**Cheng et al. 2019**	12	de novo, NA	34y	yes	no	no	global DD, severe ID	hyper	NA	excessive vomiting, GE reflux, coeliac disease	no	NA	no	epilepsy, sleep disturbance
	13	de novo, NA	13y	no	no	no	global DD, learning disability, autistic traits, pica	no	NA	gut dismotility, food intolerance	long QT	astigmatism, hyperopia	no	cutis marmorata
	14	de novo, NA	15y	no	no	no	global DD, severe ID, autism spectrum disorder	no	NA	NA	tetralogy of Fallot	NA	no	no
	15	de novo, NA	7y 6mo	bitemporal narrowing, arched eyebrows, synophris, upturned nose	yes	yes	global DD, severe ID	yes	intracranial hemorrhage, ventriculomegaly	yes	no	cortical visual impairment, astigmatism	no	partial epilepsy, VP shunt
	16	de novo, NA	1y 2mo	yes	no	no	global DD	no	no	yes	secundum ASD, mild valvular	NA	no	bilateral hearing loss

											pulmonary stenosis, dilated right atrial and ventricle			
	17	de novo, NA	6y	yes	no	yes	global DD, behavioural issues	no	NA	yes	long QT	NA	extra rib, extra vertebrae	seizures
	18	de novo, NA	2y 6mo	yes	no	yes	global DD	mild hypo	NA	yes, GE reflux	NA	astigmatism	no	no
	19	de novo, NA	7y	yes	yes	yes	global DD, severe ID, autism spectrum disorder	mild hypo, cerebral palsy	central white matter loss, thin CC	yes	NA	astigmatism, myopia	no	no
**Our Case**	20	de novo, NA	18y	mild coarse face, bitemporal narrowing, arched thick eyebrows, long eyelashes, synophrys, broad tip, anteverted nares, smooth long philtrum, thin upper lip, mild low-set ears, hirsutism	-2 SD	yes	severe DD (walking at 30 mo, absent speech), severe ID, autistic traits	hypo	cerebellar and frontal lobe atrophy, thin CC, large frontal horn ventricles	no	VSD, mild to moderate hypertrophic cardio-myopathy	myopia, astigmatism, slightly pale and broad optic disc, central colobomatous defect	large fontanels, wormian bones, hip dysplasia, delayed bone age, scoliosis, Kyphosis	Lennox-Gastaut epilepsy, sensorial neuropathy, conductive hearing loss
Total	20	19/20 (de novo)		14/20	9/20	12/20	20/20	9/20 (hypo)	9/20	15/20	9/20	13/20	10/20	

ASD, atrial septal defect; CC, *corpus callosum*; CSF, cerebrospinal fluid; DD, developmental delay; ID intellectual disability; HIE, hypoxic-ischemic encephalopathy; IVH, intraventricular hemorrhage ; MGM, maternal germline; NA, not available; PAS, pulmonary artery stenosis; PEG, percutaneous endoscopic gastrostomy; PFO, patent foramen ovale; PVL periventricular leukomalacia; VP ventriculo-peritoneal; VSD, ventricular septal defect.
